# Supplementary material for: Pyruvate Dehydrogenase Kinase 1 inhibition mediated oxidative phosphorylation enhancement in cartilage promotes osteoarthritis progression
Source: BMC Musculoskelet Disord. 2023 Jul 20;24:597. doi: 10.1186/s12891-023-06585-6 (PMC10357736; doi:10.1186/s12891-023-06585-6)
Supplement: Supplementary file 1 — Additional file 1. [file 12891_2023_6585_MOESM1_ESM.zip › MF.Gsea.1653627316242/GOMF_NUCLEOTIDE_DIPHOSPHATASE_ACTIVITY.html]

Details for gene set GOMF\_NUCLEOTIDE\_DIPHOSPHATASE\_ACTIVITY[GSEA]

|  || Dataset | 55457\_collapsed\_to\_symbols.cls.cls #OA\_versus\_normal.cls.cls #OA\_versus\_normal\_repos |
| Phenotype | cls.cls#OA\_versus\_normal\_repos |
| Upregulated in class | OA |
| GeneSet | GOMF\_NUCLEOTIDE\_DIPHOSPHATASE\_ACTIVITY |
| Enrichment Score (ES) | 0.5210407 |
| Normalized Enrichment Score (NES) | 1.5743818 |
| Nominal p-value | 0.04288939 |
| FDR q-value | 0.14791794 |
| FWER p-Value | 0.991 |
Table: GSEA Results Summary

  

Fig 1: Enrichment plot: GOMF\_NUCLEOTIDE\_DIPHOSPHATASE\_ACTIVITY      
 Profile of the Running ES Score & Positions of GeneSet Members on the Rank Ordered List

  

| SYMBOL | TITLE | RANK IN GENE LIST | RANK METRIC SCORE | RUNNING ES | CORE ENRICHMENT || 1 | NUDT11 | nudix hydrolase 11 [Source:HGNC Symbol;Acc:HGNC:18011] | 630 | 0.611 | 0.0901 | Yes |
| 2 | ENPP4 | ectonucleotide pyrophosphatase/phosphodiesterase 4 [Source:HGNC Symbol;Acc:HGNC:3359] | 822 | 0.563 | 0.2034 | Yes |
| 3 | ASMTL | acetylserotonin O-methyltransferase like [Source:HGNC Symbol;Acc:HGNC:751] | 1143 | 0.487 | 0.2895 | Yes |
| 4 | CILP | cartilage intermediate layer protein [Source:HGNC Symbol;Acc:HGNC:1980] | 1820 | 0.381 | 0.3238 | Yes |
| 5 | NUDT6 | nudix hydrolase 6 [Source:HGNC Symbol;Acc:HGNC:8053] | 2031 | 0.358 | 0.3890 | Yes |
| 6 | FHIT | fragile histidine triad diadenosine triphosphatase [Source:HGNC Symbol;Acc:HGNC:3701] | 2758 | 0.274 | 0.3951 | Yes |
| 7 | NUDT3 | nudix hydrolase 3 [Source:HGNC Symbol;Acc:HGNC:8050] | 2870 | 0.264 | 0.4464 | Yes |
| 8 | NUDT13 | nudix hydrolase 13 [Source:HGNC Symbol;Acc:HGNC:18827] | 3172 | 0.234 | 0.4762 | Yes |
| 9 | ENPP1 | ectonucleotide pyrophosphatase/phosphodiesterase 1 [Source:HGNC Symbol;Acc:HGNC:3356] | 3392 | 0.212 | 0.5075 | Yes |
| 10 | ITPA | inosine triphosphatase [Source:HGNC Symbol;Acc:HGNC:6176] | 3751 | 0.181 | 0.5210 | Yes |
| 11 | NUDT2 | nudix hydrolase 2 [Source:HGNC Symbol;Acc:HGNC:8049] | 4521 | 0.112 | 0.4867 | No |
| 12 | GARS1 | glycyl-tRNA synthetase 1 [Source:HGNC Symbol;Acc:HGNC:4162] | 7069 | -0.085 | 0.3085 | No |
| 13 | NUDT15 | nudix hydrolase 15 [Source:HGNC Symbol;Acc:HGNC:23063] | 8305 | -0.178 | 0.2533 | No |
| 14 | ENPP2 | ectonucleotide pyrophosphatase/phosphodiesterase 2 [Source:HGNC Symbol;Acc:HGNC:3357] | 8442 | -0.189 | 0.2858 | No |
| 15 | NUDT4 | nudix hydrolase 4 [Source:HGNC Symbol;Acc:HGNC:8051] | 9400 | -0.264 | 0.2716 | No |
Table: GSEA details [plain text format]

  

Fig 2: GOMF\_NUCLEOTIDE\_DIPHOSPHATASE\_ACTIVITY      
 Blue-Pink O' Gram in the Space of the Analyzed GeneSet

  

Fig 3: GOMF\_NUCLEOTIDE\_DIPHOSPHATASE\_ACTIVITY: Random ES distribution      
 Gene set null distribution of ES for **GOMF\_NUCLEOTIDE\_DIPHOSPHATASE\_ACTIVITY**
